# *Tosanoidesannepatrice*, a new basslet from deep coral reefs in Micronesia (Perciformes, Percoidei, Serranidae)

**DOI:** 10.3897/zookeys.786.28421

**Published:** 2018-10-02

**Authors:** Richard L. Pyle, Brian D. Greene, Joshua M. Copus, John E. Randall

**Affiliations:** 1 Bernice P. Bishop Museum, 1525 Bernice Street, Honolulu, Hawai‘i 96817, USA Bernice P. Bishop Museum Honolulu United States of America; 2 Association for Marine Exploration, 47-224 Kamehameha Hwy, Kaneohe, Hawai‘i 96744, USA Association for Marine Exploration Kaneohe United States of America; 3 Hawaii Institute of Marine Biology, 46-007 Lilipuna Rd, Kaneohe, Hawai‘i 96744, USA Hawaii Institute of Marine Biology Kaneohe United States of America

**Keywords:** closed-circuit rebreather, coral-reef twilight zone, mesophotic coral ecosystems, Micronesia

## Abstract

The new species *Tosanoidesannepatrice***sp. n.** is described from four specimens collected at depths of 115–148 m near Palau and Pohnpei in Micronesia. It differs from the other three species of this genus in life color and in certain morphological characters, such as body depth, snout length, anterior three dorsal-fin spine lengths, caudal-fin length, and other characters. There are also genetic differences from the other four species of *Tosanoides* (d ≈ 0.04–0.12 in mtDNA cytochrome oxidase I). This species is presently known only from Palau and Pohnpei within Micronesia, but it likely occurs elsewhere throughout the tropical western Pacific.

## Introduction

The authors have explored mesophotic coral ecosystems (MCEs; coral-reef habitat at depths of 30–150 m; also referred to as the “coral-reef twilight zone”) throughout Micronesia for decades, and have utilized advanced mixed-gas closed-circuit rebreather diving operations since 1997, which has led to the discovery of dozens of new species of fishes (Pyle 1996a, 1996b, 2000, Pyle and Copus in press). One such new species was first discovered in 2007 during an expedition to Micronesia to film the BBC documentary *Expedition Pacific Abyss* (British Broadcasting Corporation 2007), when the second author collected a specimen of *Tosanoides* Kamohara, 1953 at a depth of 115 m off Ngaruangl Atoll, Kayangel State, Republic of Palau. Although the specimen differed substantially in color from the other two species of the genus known at the time, additional specimens were needed to ensure it was a distinct and previously undescribed species. During an expedition to Pohnpei, Federated States of Micronesia in 2015 (Rowley et al. in press), the first two authors observed additional individuals of this *Tosanoides* at a depth of 148 m off the west end of Ahnd (Ant) Atoll, outside the entrance to a small cave along a vertical reef drop-off. During a subsequent expedition to Pohnpei in 2016, the second author managed to collect one large adult male and two juvenile specimens.

The specimens represent an undescribed species of the serranid subfamily Anthiadinae Poey 1861 (commonly spelled Anthiinae), within the genus *Tosanoides*. The genus currently includes four nominal species: *Tosanoidesfilamentosus* Kamohara, 1953 (type species), *T.flavofasciatus* Katayama & Masuda, 1980, *T.obama* Pyle, Greene & Kosaki, 2016, and *T.aphrodite* Pinheiro, Rocha & Rocha, 2018. Herein we describe the fourth member of the genus, *Tosanoidesannepatrice*, based on morphologic and genetic differences compared with the other known species. In addition, the species currently referred to as *Pseudanthiasfucinus* (Randall & Ralston, 1985) from the Hawaiian Islands is likely a member of the genus *Tosanoides*, and is similar in many respects to *T.aphrodite*.

At least two other undescribed species of this genus have been reported; one from the Coral Sea (Gerald R. Allen pers. comm.) and one from Rapa Nui (Easter Island) (Easton et al. 2017).

## Materials and methods

Specimens were collected with hand nets during deep dives using mixed-gas, closed-circuit rebreathers, and brought to the surface alive with the aid of a hypodermic needle to vent gas from the swim bladders. Methods for counts and measurements follow those described in Pyle et al. (2016). Gender of adult specimens based on examination of gonads. Description template and wording follow Pyle et al. (2016), modified from Katayama and Masuda (1980) for consistency.

The holotype has been deposited at the Bernice Pauahi Bishop Museum fish collection, Honolulu (**BPBM**), and paratypes have been deposited at the U.S. National Museum of Natural History, Washington, D.C. (**USNM**) and California Academy of Sciences, San Francisco (**CAS**).

Fresh tissue samples were obtained from the holotype and paratypes. DNA barcodes (cytochrome c oxidase I; COI) were sequenced following the protocol described in Copus et al. (2015). Barcode of Life Database (BOLD) identifiers for DNA sequences are presented along with museum catalog numbers for type material and non-type specimens. A neighbor-joining tree building method was used to reconstruct the phylogenetic tree using Geneious v.6.2 (Kearse et al. 2012) under the HKY model of sequence evolution, with a random seed of 920,582, 100,000 bootstrap replicates, and 50% support threshold. Estimates of genetic differences (d) were also calculated in Geneious.

## Taxonomy

### Tosanoides annepatrice sp. n.

Taxon classificationAnimaliaPerciformesSerranidae

http://zoobank.org/CACCF76F-744C-478E-B303-24674D1C433B

**Figure 1.**
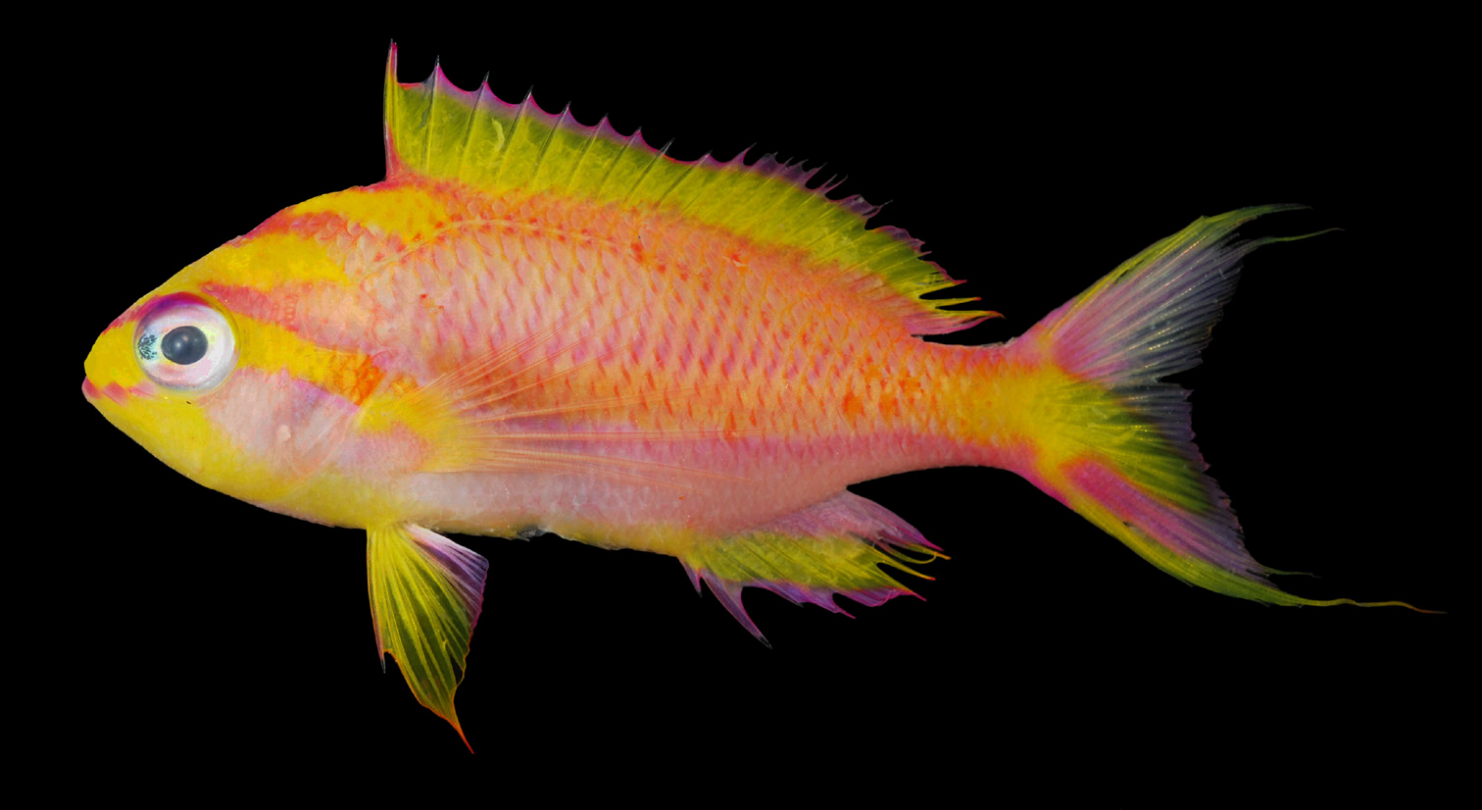
Holotype of *Tosanoidesannepatrice* (BPBM 40848), 80.9 mm TL, collected at a depth of 115 m off Ngaruangl Atoll, Kayangel State, Republic of Palau. Photograph by RL Pyle.

**Figure 2.**
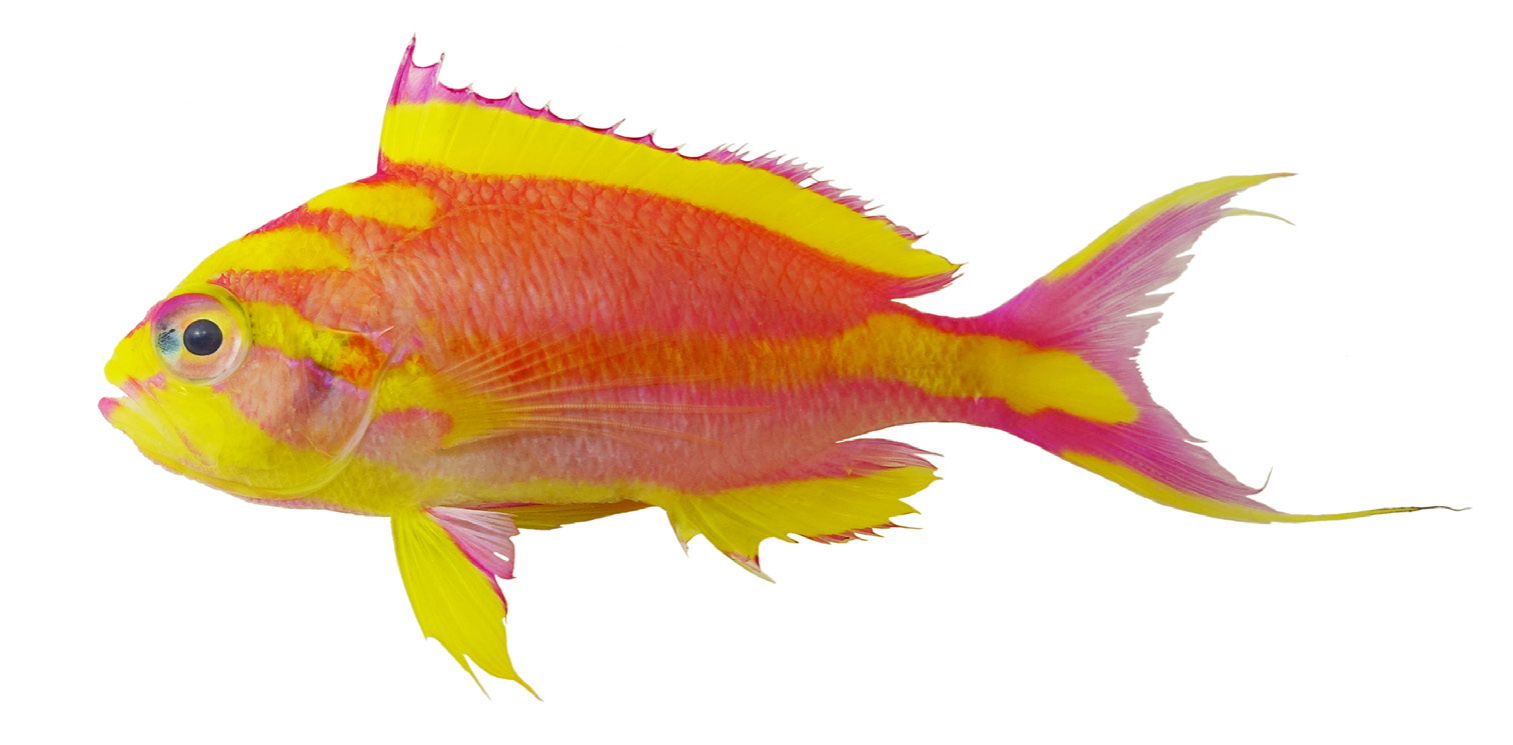
Paratype of *Tosanoidesannepatrice* (USNM 444916), 104.0 mm TL, adult male, collected at a depth of 148 m off Ahnd (Ant) Atoll, Pohnpei, Federated States of Micronesia. Photograph by BD Greene.

**Figure 3.**
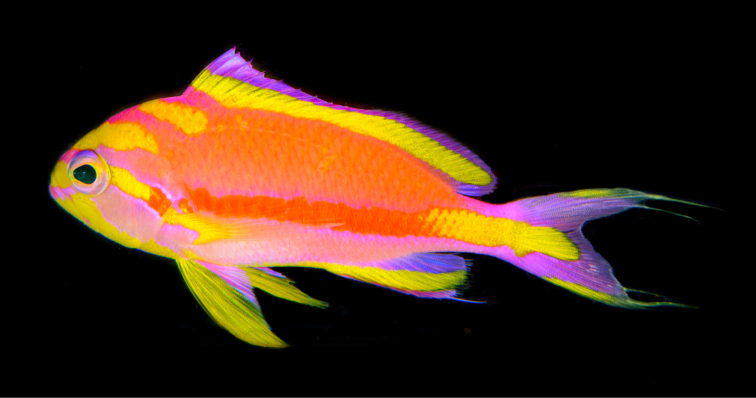
Adult male *Tosanoidesannepatrice* alive in an aquarium, collected in Pohnpei. Photograph by LA Rocha.

**Figure 4.**
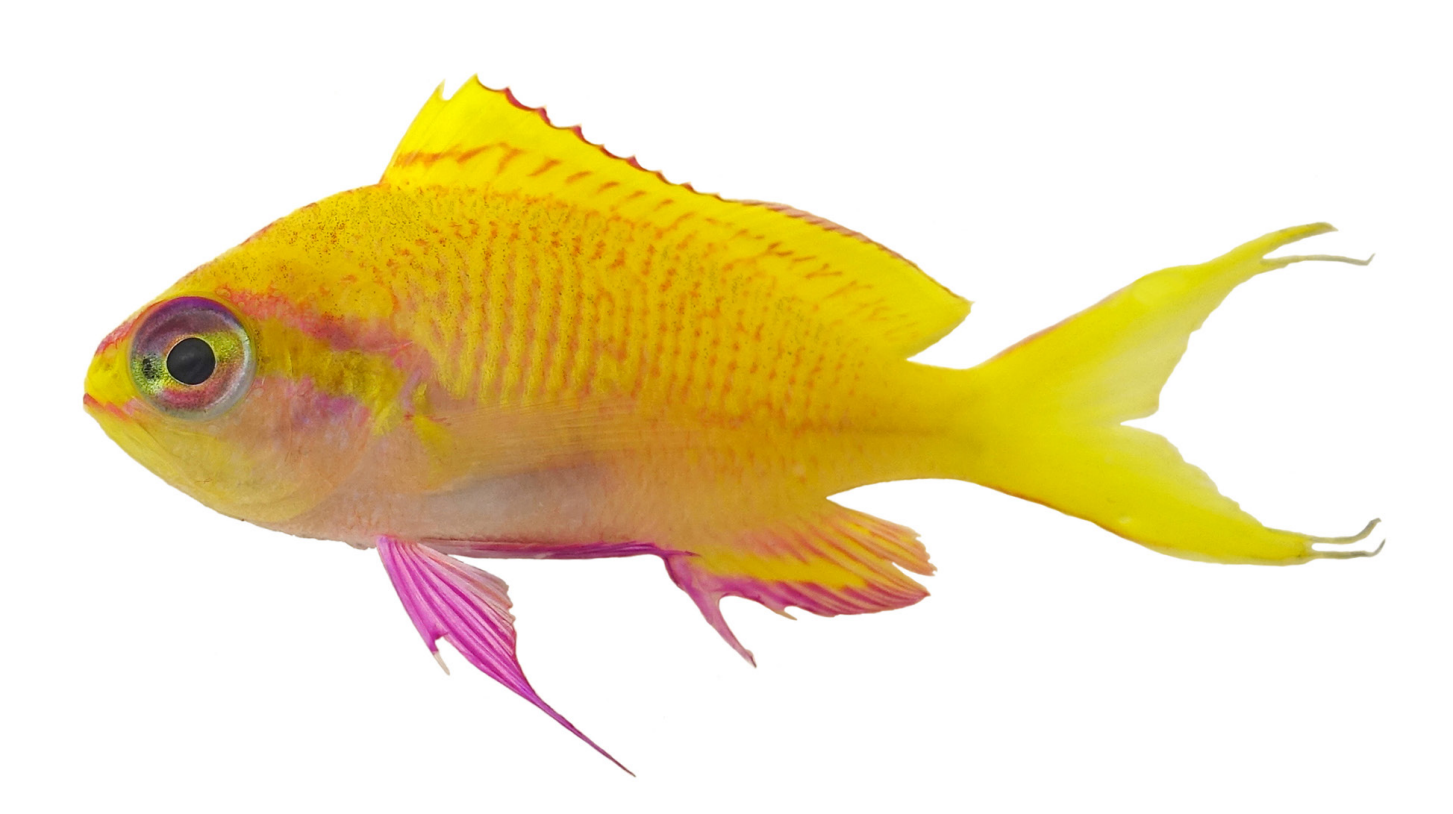
Paratype of *Tosanoidesannepatrice* (BPBM 41354), 46.8 mm TL, immature female, collected at a depth of 148 m off Ahnd (Ant) Atoll, Pohnpei, Federated States of Micronesia. Photograph by BD Greene.

**Figure 5.**
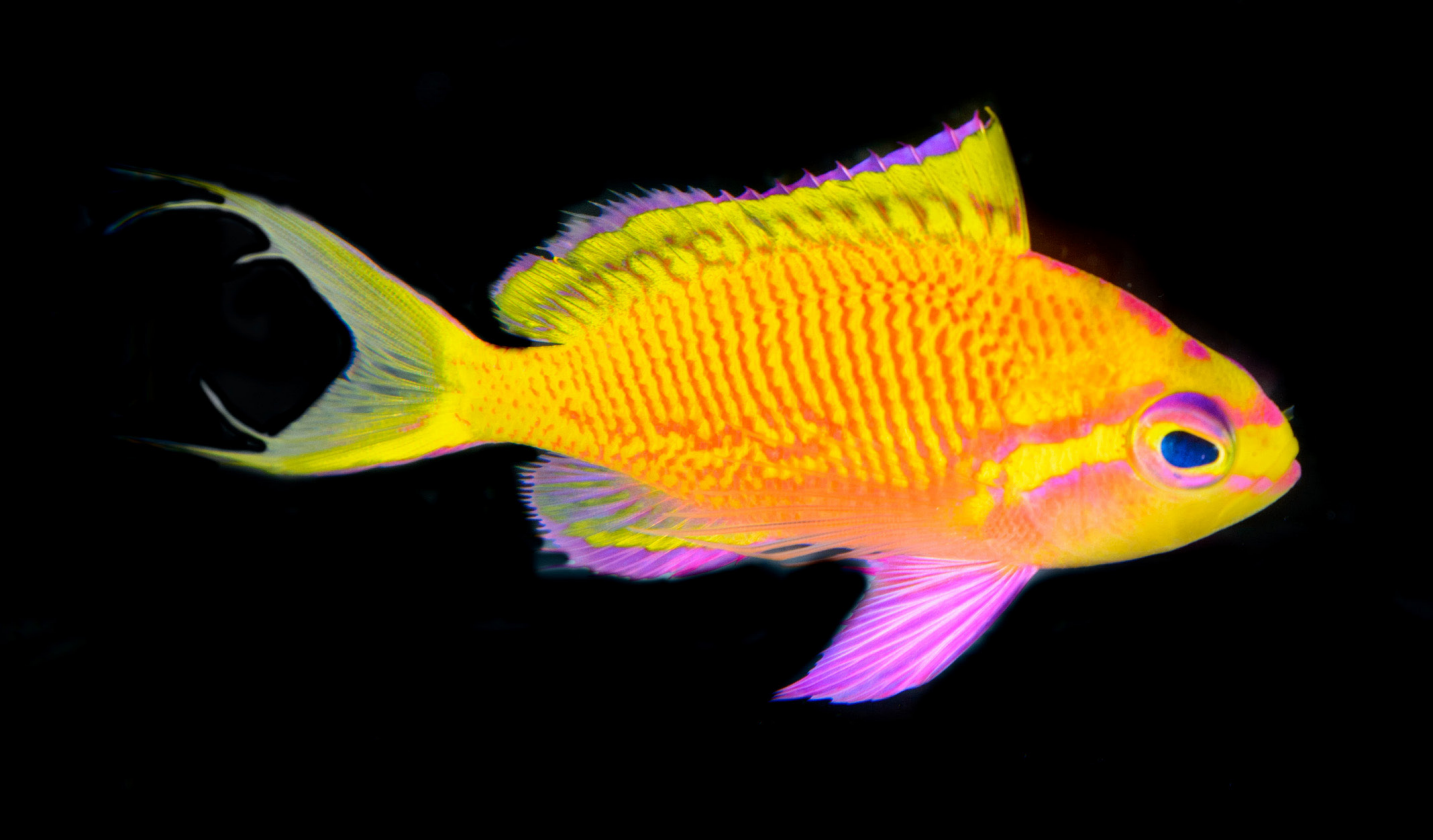
Adult female *Tosanoidesannepatrice* alive in an aquarium, collected in Pohnpei. Photograph by LA Rocha.

Figs 1 , 2 , 3 , 4 , 5

#### Type Locality.

Republic of Palau, Kayangel State, Ngaruangl Atoll, 8.14733°N, 134.61763°E.

#### Material.

**Holotype.**BPBM 40848, male, Barcode of Life TOSAN-001-18, 53.0 mm SL, Republic of Palau, Kayangel State, Ngaruangl Atoll, 8.14733°N, 134.61763°E, 115 m, 23 April 2007, B. D. Greene, aboard vessel *Big Blue Explorer*, hand nets, under rock on steep rocky slope. **Paratypes.**USNM 444916, male, Barcode of Life TOSAN-002-18, 68.7 mm SL, Federated States of Micronesia, Pohnpei, Ahnd (Ant) Atoll, W side, 6.75589°N, 157.91933°E, 148 m, 22 July 2016, B. D. Greene, hand nets, near entrance to cave on vertical drop-off.

BPBM 41354, immature, Barcode of Life TOSAN-003-18, 31.6 mm SL, same collection data as USNM 444916.

CAS 244531, immature, Barcode of Life TOSAN-004-18, 28.0 mm SL, same collection data as USNM 444916.

#### Diagnosis.

A species of *Tosanoides* (*sensu*Katayama and Masuda 1980) distinguished by the following combination of characters: fourth or fifth dorsal spine the longest; dorsal-fin soft rays 16–17; anal-fin soft rays 8; pored lateral-line scales 33–34; head 2.3–2.9 in SL; body depth 2.6 in SL; color in life of males: head and body rose-pink, crossed by four bright yellow stripes, the third continuing as a bright red stripe with magenta edges along the middle of the body, becoming yellow centered on base of caudal fin; dorsal fin with a very broad middle yellow stripe with magenta margin; base of anal fin colored like body anteriorly, grading broadly to magenta posteriorly; pelvic fins yellow, except for magenta last two rays; eye magenta with an uneven ring of yellow around pupil; color of immature and presumed female yellow with irregular, near-vertical, wavy red lines following scale margins; anal fin magenta anteriorly, grading posteriorly to purple, with a greenish yellow streak; pelvic fins bright magenta.

#### Description.

Dorsal fin X,16 (17), last soft ray branched to base; anal fin III,8, last soft ray branched to base; pectoral-fin rays 14; pelvic-fin rays I,5; principal branched caudal rays 14, upper procurrent unbranched caudal rays 6, lower procurrent unbranched caudal rays 4; pored lateral-line scales 33 (33–34); scale rows above lateral line to origin of dorsal fin 4; scale rows below lateral line to origin of anal fin 15 (14–15); gill rakers on upper limb 11, on lower limb 22 (22–23); vertebrae 26 (10+16).

Body ovoid, compressed, its greatest depth 2.62 (2.57–2.64) in SL, the width just posterior to gill opening 2.40 (2.22–2.56) in depth; head length 2.93 (2.35–2.77) in SL; snout short, its length 5.66 (4.28-7.00) in head; orbit diameter 2.41 (2.53–2.85) in head; interorbital convex, the least bony width 3.42 (3.45–5.41) in head; least depth of caudal peduncle 2.78 (2.99–3.40) in head.

Mouth large, oblique and protractile; lower jaw not projecting beyond the upper when mouth closed; maxilla 2.01 (1.92–2.20) in head, expanded distally, reaching below posterior border of pupil, slightly diagonal, the gape forming an angle of ca. 20° to the horizontal, supramaxilla absent. A pair of nostrils on either side of head, close together, directly in front of eye, anterior nostril in a membranous tube with an elevated posterior edge, posterior nostril with a slight rim anteriorly. Teeth in upper jaw villiform, forming a band broader anteriorly with a pair of canines on each side and another pair of canines slightly posteriorly and internally directed backward, an outer row of ca. ten slender canines on each side of jaw curved forward; lower jaw with a patch of villiform teeth anteriorly; one canine on each side anteriorly facing forward and a second canine on each side curved forward, an outer row of ca. 15 slender canines like those of the upper jaw, posterior ones pointing forward; small teeth on vomer and palatines, teeth on vomer in a triangular band; tongue pointed, smooth. Preopercle with a round angle, upper limb serrate with ca. 25 spinules, lower limb smooth; opercle with two flat spines, upper one longest and at apex; subopercle and interopercle smooth. Gill rakers long and numerous, with eleven rakers on the upper limb and 22 (22–23) on the lower limb, longest raker much longer than gill filament.

Dorsal fin very slightly notched, inserted slightly posterior to dorsal end of gill opening, its base 1.69 (1.65–1.73) in SL; first dorsal-fin spine the longest, 1.81 (1.97–2.32) in head, second dorsal-fin spine originating immediately posterior to first, its length 1.97 (2.00–2.48) in head, third dorsal-fin spine 2.13 (2.11–2.53) in head, fourth dorsal-fin spine, 2.29 (2.24–2.70) in head, fifth dorsal-fin spine 2.51 (2.33–2.76) in head, last dorsal-fin spine 2.83 (2.90–3.14) in head; membranes between dorsal-fin spines mildly incised; longest dorsal soft ray (seventh or eighth) 2.15 (2.58-2.78) in head. Anal fin originating below base of third or fourth dorsal soft ray; second anal spine slightly stronger than the third; length of first anal-fin spine 5.32 (5.17–6.00) in head, second anal-fin spine 2.13 (2.00–2.25) in head, third anal-fin spine 2.21 (2.19–2.59) in head; posterior margin of anal fin rounded; length of longest anal soft ray (fifth or sixth) 1.60 (1.77–2.38) in head. Pectoral fins subsymmetrical, longer than head, reaching a vertical at base of third or fourth anal soft ray, their length 2.31 (2.28–2.52) in SL; caudal fin deeply convex, upper and lower lobes each with two filamentous extensions on their outermost principle rays, its length 1.90 (1.95–2.26); pelvic spine 1.83 (1.66–2.15) in head; first soft ray of pelvic fin with a filamentous extension (broken in holotype), its length (3.07–4.08), in SL.

Scales moderately large, ctenoid; four in a series from origin of dorsal fin to lateral line, 15 (14–15) from origin of anal fin to lateral line; head closely scaled except for lips and tip of snout anterior to nostrils; dorsal and anal fins with small scales basally, a single row on spinous portion of dorsal fin, reaching distally ca. 1/5 of distance to outer margin posteriorly on soft portions of dorsal and anal fins; ca. seven or eight vertical scale rows on base of caudal fin; scales on pectoral fins basally, extending posteriorly on lower half of pectoral fin approximately one third distance to posterior margin. Lateral line with 33 (33–34) pored scales, high, concurrent with back, forming an angle below last several dorsal rays and extending along middle of caudal peduncle to base of caudal fin.

The two immature paratypes differ from the adult specimens in having a proportionally larger head (2.35–2.77 in SL, compared with 2.77–2.93), shorter snout (6.33–7.00 in head, compared with 4.28–5.66), longer dorsal-fin base (1.70–1.73 in SL, compared with 1.65–1.69) and anal-fin base (4.65–4.91 in SL, compared with 4.91–5.05), narrower caudal peduncle (3.17–3.40 in head, compared with 2.78–2.99), shorter pelvic spine (2.09–2.15 in head, compared with 1.66–1.83), longer pelvic fin (2.43–2.62 in head, compared with 3.07–4.08), shorter dorsal-fin rays (longest 2.77–2.78 in head, compared with 2.15–2.58) and anal-fin rays (longest 2.28–2.38 in head, compared with 1.60–1.77), shorter first anal-fin spine (5.95–6.00 in head, compared with 5.17–5.32), and shorter caudal-fin (2.08–2.26 in SL, compared with 1.90–1.95).

Color in life as in Fig. 1–5. This species is sexually dichromatic. Male (holotype; Fig. 1–3): head and body rose-pink, the scale edges orange-red; head crossed by four bright yellow stripes, the upper three one-third to one-half eye diameter in width, the first high on nape, ending below base of third dorsal spine, second beginning on anterior profile at upper edge of eye, curving as it passes dorsally, and narrowing to a point nearly to origin of lateral line; third starts on upper lip, covers most of side of snout, reappears behind upper half of eye, continues slightly downward to a mixed orange-yellow spot just below opercular spine, and links to a short yellow band that ends at upper half of pectoral fin, continuing as a bright red stripe with magenta edges along the middle of the body, becoming a broad oblique yellow stripe centered on base of caudal fin; fourth stripe covers most of ventral part of head, leaving only a pink tip to the lower jaw and a mark like two tiny pink flags obliquely across upper lip and suborbital; yellow stripe continues ventrally on chest and abdomen, narrowing as it passes to origin of anal fin; dorsal fin with a very broad middle yellow stripe, colored like body basally, with a magenta margin that covers outer half of first dorsal spine; anal fin mainly yellow, continuous anteriorly with yellow band ventrally on abdomen; base of fin colored like body anteriorly, grading broadly to magenta posteriorly; an orange-red submarginal line on anteriorly half of soft portion of fin; pectoral fins with hyaline membranes and yellow rays that broaden ventrally to a triangular yellow spot; pelvic fins yellow, except for magenta last two rays, the two zones separated by an orange-red line; eye magenta with an uneven ring of yellow around pupil, and a large, white-edged, blue-green spot before pupil. Immature (Figure 4) and presumed female (Figure 5) fish: head and body bright yellow with irregular, near-vertical, wavy red lines following scale margins, those above lateral line narrower, the 13 lines below soft portion of dorsal fin ending ventrally in a very irregular red stripe at level of lower edge of eye, reappearing reversely slanted ventrally; five short oblique thin red lines on side of nape; ca. nine progressively shorter and thinner broken lines on caudal peduncle and base of caudal fin, reversely slanted on lower half; lines ending in abdomen and above spinous portion of anal fin obscured by reddish violet pectoral fin; head bright yellow like body with two narrow mixed magenta-violet parallel bands, one horizontal from front of snout through upper part of eye, the second from lower lip through lower part of eye, both continuing obliquely downward across postorbital head; three bright magenta marks along upper margin of nape, the first as a spot above eye, the next two as dashes at edge of nape, the latter ending at origin of dorsal fin; dorsal fin bright greenish yellow with a bright purple margin that narrows posteriorly on spinous portion of fin, then broadens on sort portion, again narrowing posteriorly; an obscure blackish red line basally on spines and rays of dorsal fin, in height ca. equal to maximum width of pupil, some branching to irregular Y-shape; anal fin magenta anteriorly, grading posteriorly to purple, with a greenish yellow streak from base of spinous portion of fin to outer fourth of fourth-from-last anal ray; caudal fin greenish yellow basally and out on upper and lower margins, progressively narrower posteriorly, rays in rest of fin gradually changing from yellow to bluish gray posteriorly; pelvic fins bright magenta; eye with a bluish purple pupil ruined by violet, the dorsal edge with an arc of dark purple.

Color in alcohol uniformly pale yellow except for eye, which is black.

Morphometric data for selected characters of type specimens are provided in Table 1.

**Table 1.** Morphometric and meristic data for selected characters of type specimens of *Tosanoidesannepatrice*. Values of morphometric data (other than TL and SL) are represented as % of SL.

**Measurements**	**Holotype**	**Paratype**	**Paratype**	**Paratype**
	**BPBM 41315**	**USNM 444916**	**BPBM 41354**	**CAS 244531**
**Morphometrics**				
Sex	Male	Male	Immature	Immature
Total length (TL) in mm	80.9	104.0	46.8	40.4
Standard length (SL) in mm	53.0	68.7	31.6	28.0
Head length	34.2	36.1	36.1	42.5
Body depth	38.1	38.7	38.9	37.9
Body width	41.6	45.1	39.0	43.4
Snout length	17.7	23.4	15.8	14.3
Predorsal length	38.3	38.1	37.3	39.3
Preanal length	68.1	63.2	61.7	65.7
Base of dorsal fin	59.2	60.7	57.9	58.9
Base of anal fin	19.8	20.4	21.5	20.4
Orbit diameter	41.4	35.1	39.5	36.1
Interorbital width	29.3	27.4	28.9	18.5
Caudal peduncle depth	35.9	33.5	31.6	29.4
Pelvic spine	54.7	60.1	46.5	47.9
Pelvic fin	24.5	32.6	41.1	38.2
First dorsal spine length	55.2	43.1	50.9	43.7
Second dorsal spine length	50.8	43.1	50.0	40.3
Third dorsal spine length	47.0	42.3	47.4	39.5
Fourth dorsal spine length	43.6	37.1	44.7	38.7
Fifth dorsal spine length	39.8	36.3	43.0	37.8
Last dorsal spine length	35.4	31.9	34.2	34.5
Longest dorsal ray length	46.4	38.7	36.0	36.1
First anal spine length	18.8	19.4	16.7	16.8
Second anal spine length	47.0	45.2	50.0	44.5
Third anal spine length	45.3	40.7	45.6	38.7
Longest anal ray length	62.4	56.5	43.9	42.0
Caudal fin length	52.6	51.4	48.1	44.3
Pectoral fin length	43.2	39.7	42.4	43.9
**Meristics**				
Dorsal spines	X	X	X	X
Dorsal rays	16	17	17	17
Anal spines	III	III	III	III
Anal rays	8	8	8	8
Pectoral rays	14	14	14	14
Pored lateral line scales	33	33	33	34
Dorsal scale rows	4	4	4	4
Ventral scale rows	15	14	15	14
Gill rakers	11+22	11+22	11+23	11+22

#### Distribution.

*Tosanoidesannepatrice* is known on the basis of four specimens, one (the holotype) collected at a depth of 115 m in Palau, and three paratypes collected at a depth of 148 m near Pohnpei. Additional individuals have been observed at depths of ~120–150 m at Pohnpei. The species likely occurs at similar depths throughout much of Micronesia, and perhaps more broadly within the tropical western Pacific; but more exploration of habitat at appropriate depths throughout this region is necessary to determine its complete geographic range.

#### Habitat and Ecology.

*Tosanoidesannepatrice* has been observed and collected along steep limestone coral-reef drop-offs at depths from 115–150 m. The paratypes were collected along a small rocky crevice near the entrance to a cave, but other individuals have been seen in similar habitats not in association with caves. Most individuals of this species have been observed in groups consisting of one apparent male and several apparent females and juveniles.

#### Etymology.

We name this species *annepatrice* (a noun in apposition) in honor of Anne Patrice Greene, mother of Brian D. Greene who collected all known specimens of this new species, in recognition of the support and encouragement she has consistently provided to Brian’s exploration of the deep coral reefs of Micronesia.

#### Morphological comparisons.

The morphology of this species is consistent with the diagnosis for the genus *Tosanoides* as presented by Katayama & Masuda 1980. Compared with *Pseudanthias* Bleeker, 1871 (the only other genus it resembles), *T.annepatrice* shares with the other three species of *Tosanoides* fewer pored lateral-line scales (30–34, compared with 35–52) number of anal soft rays (8, compared with 6–7), and unbranched pectoral fin rays. Morphological comparisons with other species of *Tosanoides* are as follows, and only compare values for adult specimens of *T.annepatrice*.

**Figure 6.**
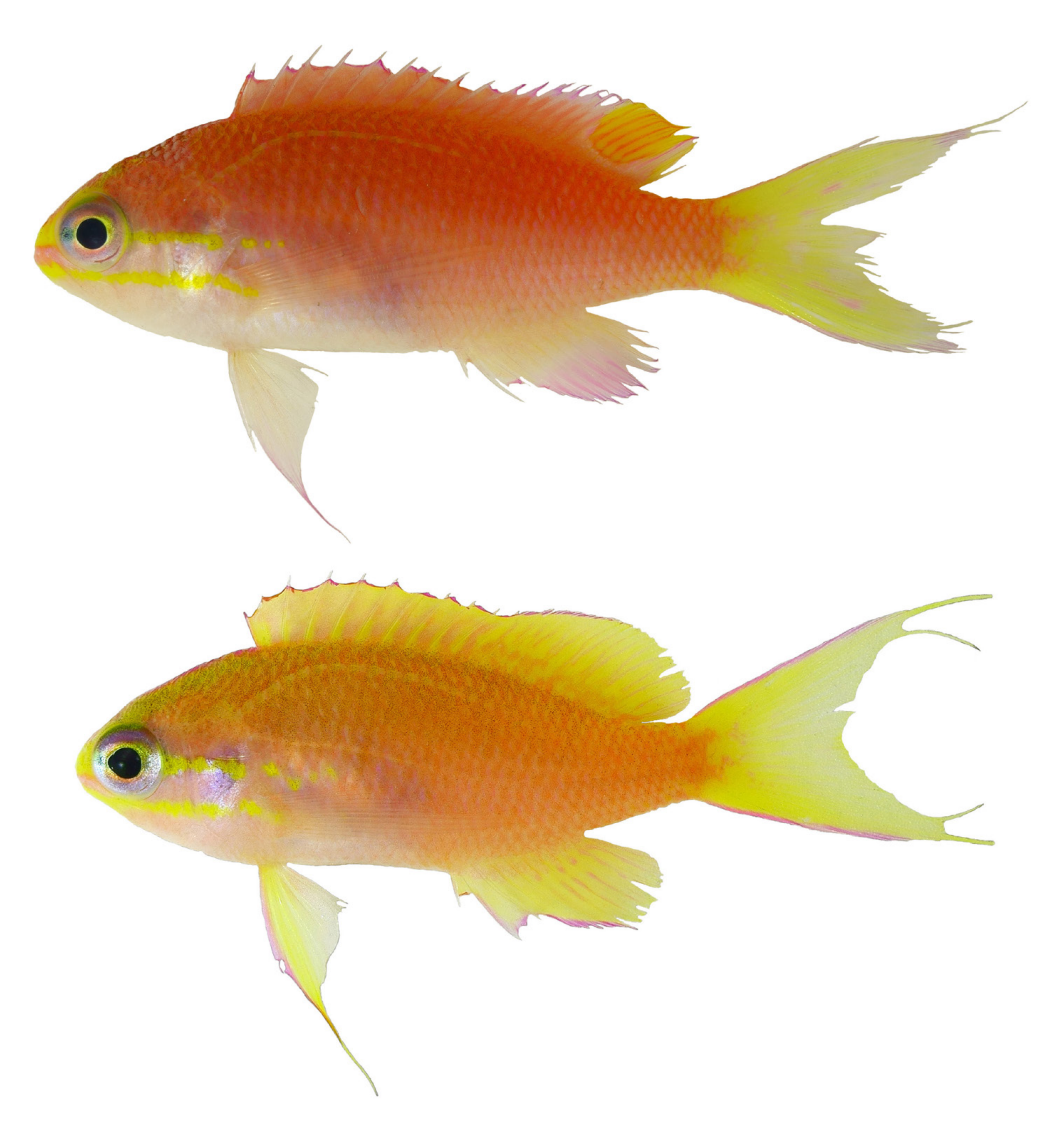
Holotype (upper; BPBM 41315) and paratype (lower; USNM 440451) of *Tosanoidesobama* collected at depths of 90–92 m in the Northwestern Hawaiian Islands. Photographs by RL Pyle.

**Figure 7.**
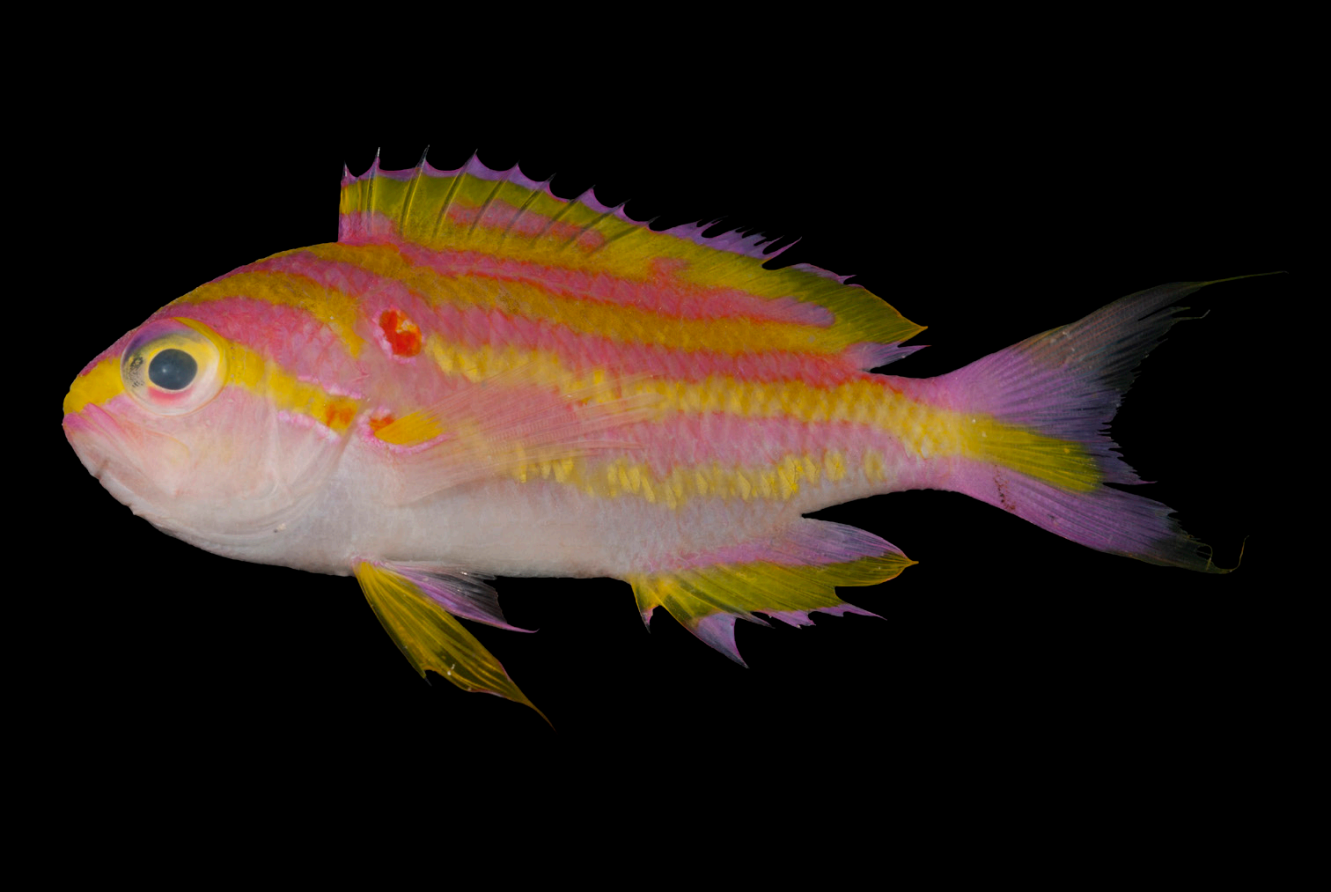
*Tosanoidesflavofasciatus*, BPBM 40858, collected at a depth of 120 m off Ngemelis Island, Republic of Palau. Photograph by RL Pyle.

*Tosanoidesannepatrice* is more similar morphologically to *T.filamentosus* than to *T.obama* (Figure 6) or *T.flavofasciatus* (Figure 7), primarily on the basis of proportional dorsal-fin spine lengths (first dorsal spine the longest, compared with third or fourth dorsal spine the longest in *flavofasciatus*), body depth (2.6 in SL, compared with 2.5 for *T.flavofasciatus* and 2.8–2.9 for *T.obama*), and snout length (4.3–5.7 in head, compared with 2.3–2.9 for *T.flavofasciatus* and 6.4 – 7.1 for *T.obama*). It is also similar to *T.obama* in number of pored lateral line scales (33–34, compared with 30–32 for *T.flavofasciatus* and 35 for *T.filamentosus*) and number of pectoral-fin rays (14, compared with 13 for *T.flavofasciatus* and *T.filamentosus*). The dorsal-fin profile of *T.annepatrice* differs considerably from both *T.flavofasciatus* and *T.obama*, in having much longer spines (2.6 in head, compared with 2.7–3.0). Another unique feature of *T.annepatrice* is that in both adult specimens, the second dorsal-fin spine is immediately adjacent to the first spine. This feature is not evident in the two immature paratypes, so it is uncertain whether this character is typical of all adults, only adult males, or is peculiar to these two specimens.

All four species of *Tosanoides* can also be easily distinguished from each other on the basis of life color.

#### Genetic comparisons.

**Figure 8.**
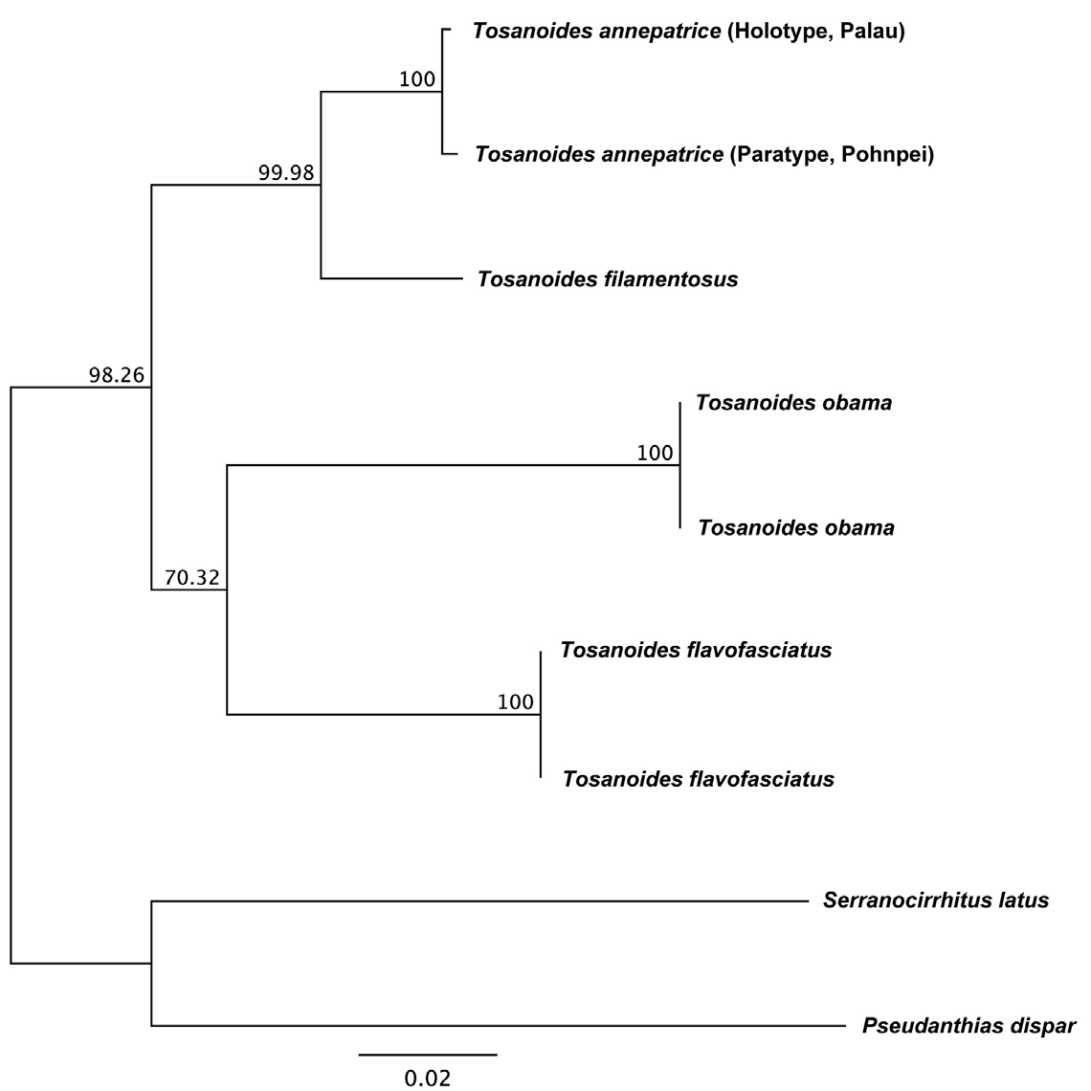
Neighbor-joining phylogenetic reconstruction of *Tosanoides* rooted with outgroups *Serranocirrhityslatus* and *Pseudanthiasdispar*. Node labels represent percent consensus support for each node. Compiled by Joshua M Copus.

Vertebrate mtDNA barcode (cytochrome oxidase I) sequences obtained from *T.annepatrice* reveal genetic differences of 3.9–4.0% when compared with *T.filamentosus*, and 9.9–12% pairwise genetic distances when compared with the other two described species of *Tosanoides* (Figure 8). The difference from *T.filamentosus* is consistent with many species-level divergences in other fish taxa (e.g., Johns and Avise 1998, Bellwood et al. 2004, Fessler and Westneat 2007, Randall and Rocha 2009, Rocha 2004, Rocha et al. 2008, Pyle and Kosaki 2016), and considerably more so for the other two species. Based on a preliminary genetic analysis, all four species of *Tosanoides* have closer genetic affinities to each other than to representatives of other Indo-Pacific anthiadine genera analyzed (Figure 8). On this basis, as well as morphological comparisons, we are confident in assigning the new species to the genus *Tosanoides* until a more exhaustive investigation of phylogenetic relationships among the species in this subfamily is completed.

#### Discussion.

*Tosanoidesannepatrice* is yet another example of several new fish species that have been discovered on deep coral reefs over the past several decades, mostly involving the use of modern mixed-gas closed-circuit rebreather diving technology (Pyle 1996a, 1996b, 2000, in press). In recent years there has been increased attention focused on mesophotic coral ecosystems (MCEs), coral-reef habitat at depths of approximately 30–150 m in tropical regions worldwide (Hinderstein et al. 2010, Baker et al. 2016, Loya et al. in press). Many more new species of fishes and other reef-associated marine organisms are likely to be discovered as a result of continued exploratory work in this poorly documented environment (Pyle and Copus in press, Pyle et al. in press), and particularly throughout Micronesia (Rowley et al. in press).

## Supplementary Material

XML Treatment for Tosanoides annepatrice
